# Comparison of human uterine cervical electrical impedance measurements derived using two tetrapolar probes of different sizes

**DOI:** 10.1186/1475-925X-5-62

**Published:** 2006-11-24

**Authors:** Saurabh V Gandhi, Dawn C Walker, Brian H Brown, Dilly OC Anumba

**Affiliations:** 1 Academic Unit of Reproductive and Developmental Medicine, University of Sheffield & Sheffield Teaching Hospitals NHS Trust, Sheffield, UK; 2 Department of Computer Science, University of Sheffield, Sheffield, UK; 3 Department of Medical Physics and Engineering, University of Sheffield, Sheffield, UK

## Abstract

**Background:**

We sought to compare uterine cervical electrical impedance spectroscopy measurements employing two probes of different sizes, and to employ a finite element model to predict and compare the fraction of electrical current derived from subepithelial stromal tissue.

**Methods:**

Cervical impedance was measured in 12 subjects during early pregnancy using 2 different sizes of the probes on each subject.

**Results:**

Mean cervical resistivity was significantly higher (5.4 vs. 2.8 Ωm; p < 0.001) with the smaller probe in the frequency rage of 4–819 kHz. There was no difference in the short-term intra-observer variability between the two probes. The cervical impedance measurements derived *in vivo *followed the pattern predicted by the finite element model.

**Conclusion:**

Inter-electrode distance on the probes for measuring cervical impedance influences the tissue resistivity values obtained. Determining the appropriate probe size is necessary when conducting clinical studies of resistivity of the cervix and other human tissues.

## Background

Electrical impedance Spectroscopy (EIS) has been employed to study the respiratory, gastro-intestinal, and cardio-vascular systems [1]. Recently, EIS has been used to study human uterine cervical tissue both *in vitro*[2,3] and *in vivo *in nonpregnant [4,5] and pregnant [6-8] women. The electrical properties of biological tissues are a function of the electrical frequency applied, and the resistive and charge storage (capacitive) properties of cellular and non-cellular components of tissues. Results from previous finite element (FE) models suggest that at low frequencies cervical impedance is high, principally from epithelial tissue, whereas at mid and higher frequencies the impedance is influenced by both superficial and sub-epithelial, stromal tissues [3]. Data on such tissue impedance may prove of clinical utility in assessing tissue health and disease.

The human uterine cervix is a common site of gynaecological malignancy. The cervix also serves key functions during human pregnancy; retaining the conceptus within the uterus until term when dramatic changes characterised by softening, shortening and dilatation eventually lead to fetal expulsion during parturition. We are currently investigating cervical epithelial (in relation to precancer) and stromal (in relation to cervical prelabour ripening and preterm labour) resistivity. Using a 5 mm probe, and measuring at eight electrical frequencies between 4.8 kHz and 614 kHz, Brown et al [4] reported significant differences in cervical resistivity between subjects with normal epithelium and those with histologically-proven cervical intra-epithelial neoplasia. Employing the same probe, one study [7] reported significant differences in cervical impedance between pregnant and non-pregnant women at a single frequency of 4.8 kHz. A subsequent study employed a larger probe of 8 mm diameter and reported differences in cervical resistivity between women with clinical cervical parameters judged favourable for induction of labour and another group judged unfavourable [6]. This study suggested a significant positive correlation between cervical impedance and the time interval from induction of labour to delivery.

Most recently, we have described cervical impedance using a 9 mm probe in a cohort of non-pregnant and pregnant women at various gestations, and noted an increase in resistivity in the third trimester perhaps resulting from changes in the collagen content and cervical cellular infiltration preparatory to prelabour ripening [8]. We obtained measurements at 30 different frequencies and determined that the cervical impedance differed the most between pregnant and non-pregnant women, and was least variable, in the frequency range 4–819 kHz.

Studies employing a wider probe are informed by the general principle that for a uniform, isotropic material, increasing the inter-electrode distance on the impedance probe will increase the depth of penetration of the electrical current. Therefore, increasing inter-electrode distance will increase the relative sensitivity at a given depth and hence provide more information about the remodelling of deeper cervical stromal structures during the process of prelabour cervical ripening. We have previously applied FE modelling – a computational technique that can be used as an aid in prediction and interpretation of *in vivo *electrical impedance spectra [3] – to study current distribution with depth in normal and malignant cervical tissue. Such modelling employs a knowledge of the structural details of a tissue comprised of different components, with known electrical properties, to predict the pattern of current flow and the measured impedance spectrum for that tissue [2,3,9]. These results have suggested that in a highly structured tissue incorporating relatively high cell density (and hence high impedance at low frequencies) epithelium, located between a relatively low impedance surface mucus layer and stroma, this simple approximation cannot be applied [3]. Hence, the relative sensitivity to structural features of the tissue measured with probes incorporating different electrode sizes and spacings remains uncertain.

The objectives of this study were to: (a) compare cervical impedance (CI), measured in a group of subjects, employing two probes measuring 5 mm and 9 mm in diameter, (b) compare the intra-observer variability of measurements obtained by the two probes, and (c) predict the fraction of current flow that is likely to be stromal (as opposed to epithelial) for both probes, using a FE model, which will indicate relative depth sensitivity.

## Methods

We measured cervical impedance in 12 healthy women undergoing pregnancy termination during the first trimester. All subjects gave written informed consent and the study was approved by the South Sheffield Research Ethics Committee. Women with previous cervical surgery or a recently abnormal cervical smear were excluded. Women were studied whilst under general anaesthetic but prior to their surgical termination of pregnancy.

Cervical impedance was measured as previously described [8]. Subjects were placed in the dorsal position and a vaginal speculum used to expose the cervix. To eliminate inter-observer variability, all the measurements were done by a single researcher. The measuring probe was then gently placed on the anterior lip of the ectocervix at the 12 o'clock position. The resulting impedance spectral data, shown graphically on the computer monitor, was then captured for analysis. Two measurements 1–2 minutes apart were taken from each subject using each of both probes. The mean of the two measurements at each frequency was employed for data analysis. These two measurements were also used to derive short term intra-observer variability for the index probe. This was determined by obtaining the mean difference in mean CI (4 to 819 kHz) between the 2 measurements for the 12 subjects with each probe and comparing these by analysis of variance. The order of the application of the probes was random to obviate systematic measurement errors.

The basic design of the cervical impedance measurement system *in vivo *has been described previously [4,8]. 5 mm (inter-electrode distance 2.2 mm) and 9 mm (inter-electrode distance 3.9 mm) probes were connected, one after another, to a single channel Electrical Impedance Measurement System (Medical Physics and Engineering, University of Sheffield) and linked to a computer with a Matlab^® ^software interface (The Mathworks Inc. Natick, MA, USA) for data capture and display. The 5 and 9 mm probes incorporate electrodes of 1 mm and 1.6 mm diameter respectively. The impedance meter drives a current of 10 μA through the tissues via an adjacent pair of the four electrodes and then measures the 'real' part of the resulting potential via the two remaining electrodes. Each measurement obtains the impedance of the tissue at 30 different electrical frequencies ranging from 2 to 1625 kHz. The tissue impedance is displayed on a computer chart on the y-axis against the electrical frequencies in kHz on the x-axis. To check the functionality of the equipment, calibration was always performed beforehand by placing the probe in a saline solution of known electrical resistivity, with results expressed as a resistivity in Ohm metres (Ωm). The CI measurements from two probes were derived using the calibration files specific for each probe. This form of calibration, performed using saline which is both uniform and purely resistive, can only provide an 'apparent resistivity' when the probe is placed on human tissue which is inhomogeneous and electrically complex. When we use the word 'resistivity' in the text we refer to an 'apparent resistivity'.

Each measured impedance spectrum was fitted to the Cole equation [10] using a least squares minimisation technique [11] We compared the mean resistivity with both probes in the frequency range 4–819 kHz. The student t and the Mann Whitney U tests were used for statistical comparisons as appropriate.

We used our pre-existing FE model for normal cervical tissue [3,8] to estimate and compare the proportion of electrical current which goes through cervical stromal, as opposed to epithelial, tissue with the 5 mm and the 9 mm probe electrode configurations. Briefly, our model incorporates a series of consecutive layers, representing cervical stroma, different layers within the epithelium (the electrical properties of which are derived from more detailed FE models which explicitly include cellular morphology and arrangements), and finally, a thin, conductive layer representing surface mucus. As there is no reliable data on the thickness of such a layer, we have used an arbitrary value of 50 μm.

## Results

**Figure 1:**
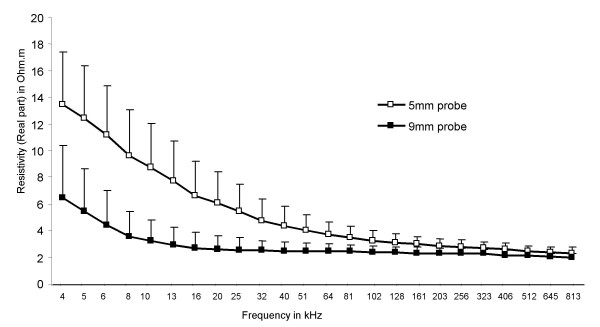
Mean (SD) impedance spectral data obtained in 12 subjects using the 5 and the 9 mm probe over frequency range 4–819 kHz (p < 0.001).

The median (range) age of the 12 study subjects was 19.6 (15–26) years. The median gestational age at the time of study was 11 (8–12) weeks. Ten patients were nulliparous and 2 had had one previous vaginal delivery. There was no difference in short term variation of measurements obtained with the two probes (Table 1). Mean coefficient of variation for the subjects studied was 31% and 34% for the 5 and 9 mm probes respectively. The mean cervical tissue resistivity in the frequency range 4–819 kHz was significantly lower with the 9 mm compared to the 5 mm probe (Table 1). This observation was most pronounced at low electrical frequencies (at 4 kHz, mean cervical resistivity 6.4 ± 3.9 vs 13.5 ± 3.9 Ω.m respectively, P < 0.01) whilst there was less difference in tissue resistivity at the higher frequencies (at 819 KHz mean cervical resistivity 2.0 ± 0.27 vs 2.26 ± 0.46 Ω.m respectively, P < 0.05, Fig 1).

**Table 1:** Mean (SD) cervical impedance data in Ωm obtained from 12 subjects, 5 mm vs. 9 mm tetrapolar measuring probe.

	***5 mm probe***	***9 mm probe***	***p value***
Mean cervical impedance over the frequency range 4–819 kHz (Ωm)	5.4 (1.6)	2.8 (0.8)	< 0.001
Mean (95% CI) difference in resistivity values obtained by single observer taking two measurements 1–2 minutes apart.	0.03(-0.8, 0.9)	-0.18 (-1.1, 0.7)	0.69

**Figure 2:**
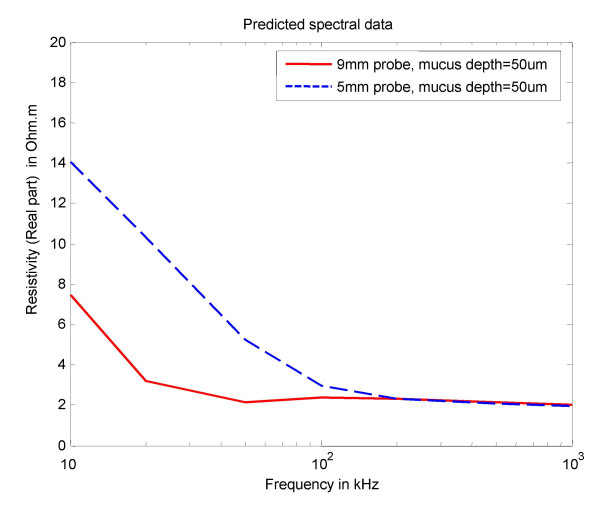
FE model predicted spectral data using the 5 and the 9 mm probe.

**Figure 3:**
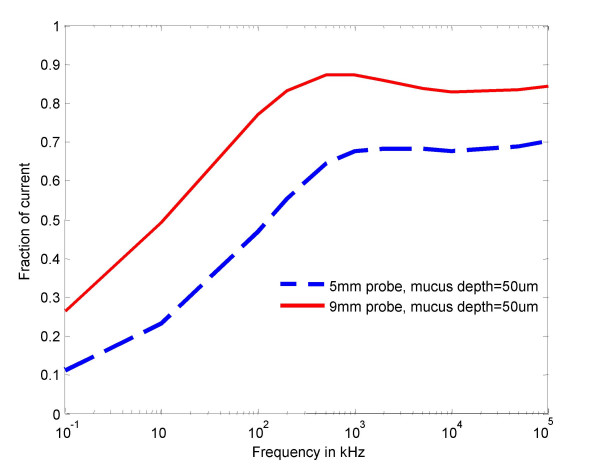
FE model prediction of stromal contribution to cervical impedance for 5 and 9 mm probes, shown as fraction of injected current flowing through cervical tissue stroma.

The computational model predicted resistivity curves for the 5 and 9 mm probes are shown in Fig 2. Predicted stromal contribution to cervical impedance for both the 5 and 9 mm probes, shown as a fraction of injected current flowing through cervical tissue stroma, is illustrated in Fig. 3.

## Discussion

This study has shown that mean cervical tissue resistivity obtained with 5 mm and 9 mm EIS probes differs significantly in the frequency range 4–819 kHz. This difference was most marked at lower electrical frequencies, with the magnitude of tissue impedance values being twice as high with the small, compared to the larger, probe. However, at higher frequencies the resistivity values obtained were similar for both probes. Short-term intra-observer variability of cervical resistivity measurement did not differ between the two probes. We noted a close correlation between our in vivo observations and the predicted cervical resistivity using a FE cervical tissue computer model. Additionally, this model suggested that the fraction of injected current passing through the cervical stroma would be higher at all frequencies for the 9 mm compared to the 5 mm probe, and would be a maximum at about 100 kHz when approximately 90% stromal penetration was attained with the larger probe, compared to about 70% penetration with the 5 mm probe.

The underlying tissue characteristics summarised in the derived resistivity spectrum for a tissue are complex and not fully explained. Tissue resistivity as captured with different probes is likely to be influenced not only by the physical and electrical properties of the probes themselves (although this is minimised by calibration), but also by the intrinsic characteristics of the tissue studied. The resistivity spectrum obtained by EIS is influenced by such tissue properties as the surface mucus, structural changes in epithelium (such as stretching, nuclear-cytoplasmic ratio, cell orientation and the associated changes in extracellular volume), and the stromal tissue characteristics [3,12-14]. Stromal contribution to tissue resistivity is influenced by such factors as extracellular hydration, matrix content and cellular density, as recently demonstrated using computational modelling [14]. Larger probes are designed to have a wider distance between the injecting and sensing electrodes. The higher the inter-electrode distance associated with large diameter probes, the deeper the current penetration into stromal tissue and therefore the greater the relative contribution of stromal elements to the obtained resistivity spectrum. We believe that the difference in the depth of penetration of electrical current into cervical tissue accounted principally for the different cervical resistivity values obtained with the 5 mm and 9 mm probes in the same group of women.

However, the relationship between electrode spacing and measured resistivity is complex, due to the anisotropy in electrical properties that arises from the highly stratified nature of the tissue. Our computer modelling suggests that at low frequencies the tissue is still stratified and this draws the current into the higher conductivity stromal tissue. However, at high frequencies current penetrates the cell membranes and the tissue appears to be homogeneous so that the relative amount of current in the stroma is reduced. These two competing effects give rise to a frequency where current penetration is greatest. In principle it is possible to compute a full sensitivity analysis for the tissue strata but this would require a much larger and more realistic FE model of the tissues to be available.

The relative proportion of current flowing in the epithelial layers and the stroma will also vary according to the probe size. With the smaller electrode array, a greater proportion of current would flow through the shallower and more resistive epithelial layers. This could explain the higher resistivitymeasured for the 5 mm probe. As the frequency increases, cell membranes become progressively more 'invisible' to current, so the electrical properties (and hence measured impedance) converge towards the higher end of the frequency range, irrespective of the current distribution.

Our study is the first to compare uterine cervical tissue resistivity obtained in a single group of subjects using two different probes. Our observations are consistent with two previous reports, obtained from two separate groups of pregnant women, which show a lower cervical resistivitymeasurement with a 5 mm compared to an 8 mm probe at 4.8 kHz frequency [6,7]. Using a 5 mm probe, O'Connell et al reported median resistivity values of 10.01 Ωm in a group of pregnant women [7], compared to mean resistivity values of 7.03 and 5.34 Ωm obtained with an 8 mm probe in two groups of pregnant women distinguished by an "unfavourable" or a "favourable" cervix for labour inducibility respectively [6]. Although these two studies reported median and mean resistivity values respectively, previous reports on cervical epithelium employing the 5 mm probe described a difference between mean and median values of resistivity of only 5% [4], suggesting that the resistivity differences between 5 and 8 mm probes noted in the papers during human pregnancy are significant. Taken together, the larger diameter probes appear to pass current more deeply into cervical tissue and are likely to be more sensitive to the lower resistivity of cervical stroma.

It may be suggested that the differences in cervical resistivity observed with the two different probes were related to differences either in the voltage delivery to the tissues by the injecting electrodes, and/or in capture of resistivity data by the sensing electrodes. This is highly implausible for several reasons. Firstly, adjustments had been made to minimise these potential differences during the design process of the probes. Secondly, both probes were calibrated in the same saline solution and device-independent cervical impedance values then derived as absolute values of resistivity in Ωm. Variations in the applied pressure of the probe on the cervix as well as the shape of the tissue at the point of application can affect the measured CI. In our study, however, this variation is likely to be minimal as all the measurements were obtained by a single researcher using subjectively similar force on the probe, and applying the probe in the same area of the anterior lip of the cervix. However, because of the difference in area of the two probe tips the pressure applied with the larger probe may be less. It is possible that this might have affected the measurements, although the difference is likely to be small as at low pressures the affect on measurements is much less than at high pressures. The similarity in coefficient of variation for both probes suggests that any difference in short-term variability between both probes is likely to be small and insignificant.

Our observations are likely to prove relevant when choosing the appropriate probe for the investigation of cervical epithelial or stromal tissue. Several studies have shown that cervical epithelial assessment of preinvasive cancer is best undertaken using a small probe which enhances spectral separation of normal from premalignant cervical epithelium [4,5]. Cervical prelabour changes are presumed to occur mainly in the stroma. If this were the case, our observations would suggest that the wider probe would prove more applicable for the study of the pregnant cervix. There is a paucity of histological evidence for this assumption, however, and there are emerging reports that changes in cervical stroma during pregnancy may be paralleled by changes in cervical epithelium. One study in mice has shown that the increase in the expression of the glycosaminoglycan hyaluron which is associated with cervical prelabour ripening not only occurs in the cervical stroma but also, to a lesser extent, in the epithelium[15]. Studies of human and animal epithelial tissue have highlighted the physiological role of gap junctional proteins such as occludin and the claudins in modulating cell contact and permeability [16-19]. Little is known of the epithelial changes associated with human cervical preparation for birth and changes in these gap junctional proteins have not been described in the cervix during human pregnancy. Insight into these changes is likely to facilitate interpretation of cervical tissue resistivity data and the design of the appropriate probe for measuring and interpreting cervical resistivity changes associated with pregnancy and prelabour that may have clinical correlates.

## Conclusion

In the final analysis, the probe characteristics which are most likely to give a measurement of prelabour inducibility or predisposition to preterm birth are ones that maintain sensitivity both to changes in stromal hydration and to structural stromal and epithelial changes that characterise these clinical events. Our studies suggest that the distance between the electrodes on probes for measuring cervical impedance influences the tissue resistivity values obtained. Consequently we suggest that determining the appropriate size of the probe is necessary when conducting clinical studies of impedance of the cervix uteri or other human tissues.

## Competing interests

The author(s) declare that they have no competing interests.

## Authors' contributions

SVG carried out the clinical experiments, participated in data analysis and drafted the manuscript. DCW carried out the finite element modelling, and participated in writing the manuscript. BHB participated in the study design and contributed to writing the manuscript. DOCA conceived of the study, participated in its design and coordination, and performed the statistical analysis. All authors read and approved the final manuscript.
